# Next step of ‘epileptic heart’

**DOI:** 10.1093/eurheartj/ehad809

**Published:** 2023-12-19

**Authors:** Yao Lu, Jie Wang, Qingwei Yu

**Affiliations:** Clinical Research Center, The Third Xiangya Hospital, Central South University, 138 Tongzipo Road, Changsha, Hunan 410013, China; Faculty of Life Sciences & Medicine, King’s College London, 150 Stamford Street, London SE1 9NH, UK; Clinical Research Center, The Third Xiangya Hospital, Central South University, 138 Tongzipo Road, Changsha, Hunan 410013, China; Clinical Research Center, The Third Xiangya Hospital, Central South University, 138 Tongzipo Road, Changsha, Hunan 410013, China; Department of Neurosurgery, Xiangya Hospital, Central South University, 87 Xiangya Road, Changsha, Hunan 410008, China

**This commentary refers to ‘Epilepsy and long-term risk of arrhythmias’, by J. Wang *et al.*, https://doi.org/10.1093/eurheartj/ehad523 and the discussion piece ‘Epilepsy and the heart: can ‘brain arrhythmia’ lead to cardiac arrhythmias? Back to the basics’, by G. L. Fialho, https://doi.org/10.1093/eurheartj/ehad807.**

We thank Dr Fialho for their discussion of our paper.^1^ People with epilepsy consistently exhibit a higher prevalence of cardiac comorbidities, recognized as a crucial factor contributing to premature death. Repeated surges in catecholamines and hypoxaemia during epileptic seizures as well as the adverse effect of antiseizure medications can lead to electrical and mechanical cardiac dysfunction, which is the concept of ‘epileptic heart’ proposed by Verrier *et al*.^2^ In addition, autonomic alterations or higher sympathetic tone in epilepsy also lead to the imbalance of cardiovascular function through the brain–heart axis. Our paper further extended the understanding of the concept that people with epilepsy have a higher long-term risk for major cardiac arrhythmias. People with epilepsy need to improve careful heart rhythm monitoring and management to mitigate the sudden cardiac death risk and long-term risk of cardiac dysfunction.

As indicated by Dr Fialho and other researchers,^3,4^ there is an increased prevalence of electrical alterations in people with epilepsy, including P-wave heterogeneity, T-wave alternans, and QT prolongation, which might be predictive of the risk of sudden cardiac death. These findings suggested that the index reflecting the cardiac electrical instability could be used in the clinical routine monitoring of people with epilepsy. Changes in cardiac structure in people with epilepsy, such as myocardial structural disarray, fibrosis, and degeneration, also imply a huge hit on the heart induced by epilepsy, which has been proved by post-mortem from the 1970s.^1^ Based on the above encouraging findings, further large-scale and well-designed cohort studies are needed to confirm these electrical markers and structural alterations, and identify additional reliable novel indicators, enhancing the understanding of epileptic heart and providing new insights for the prediction of cardiac dysfunction in people with epilepsy.

Furthermore, a more intensive understanding of the brain–heart axis is required to explain the role of anatomical and functional connections between the brain and heart. Pathophysiologic mechanisms exploration of the development of epileptic heart should be encouraged. Regarding the sudden unexpected death in epilepsy caused by cardiac arrhythmias, and higher long-term arrhythmias risk observed in our findings, the monitoring, prevention, and therapy of cardiac arrhythmias are urgent for people with epilepsy (*Figure 1*). From the three-requirement theory of cardiac arrhythmia proposed by Dr Coumel,^5^ future investigations should also focus on the substrate (control or even reverse the cardiac structure alterations, etc.), trigger (suppress seizure episode, etc.), and modulator (rectify the autonomic imbalance, etc.).

**Figure 1:**
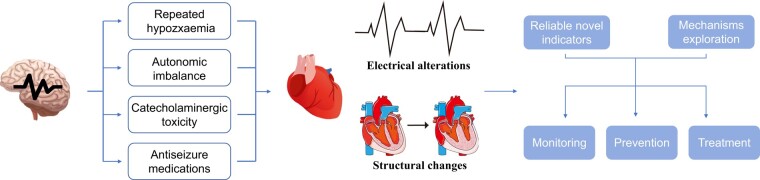
The next step of cardiac health care in people with epilepsy. People with epilepsy are predisposed to electrical alterations and structural changes in the heart that may culminate in sudden death. Reliable novel indicators, mechanisms exploration, monitoring, prevention, and treatment would be the focused aspects of the cardiac health care of ‘epileptic heart’

## Declarations

### Disclosure of Interest

All authors declare no disclosure of interest for this contribution.
